# Author’s Response to Peer Reviews of “Development of a Conversational Artificial Intelligence–Based Web Application for Medical Consultations: Prototype Study”

**DOI:** 10.2196/83417

**Published:** 2025-10-15

**Authors:** Jorge Guerra Pires

**Affiliations:** 1 IdeaCoding Lab, Rua Timbopeba, 24, Ouro Preto, 35411000, Brazil, 55 31988624279

**Keywords:** artificial intelligence, ChatGPT, chatbots, conversational agent, machine learning

*This is the author’s response to peer-review reports for “Development of a Conversational Artificial Intelligence–Based Web Application for Medical Consultations: Prototype Study.”*

## Round 1 Review

### Anonymous [1]

#### Specific Comments

##### Major Comments

*1. Please strongly consider rechecking the grammar for the paper [2].*

**Response:** Sure, done! I have checked several times and fixed typos. Please accept my apologies for those typos.

*2. Because the experience is integrated into a chatbot, indicating that providing an interactive user experience was a goal of this work, it would be good to also conduct some user research to assess the usability of the system and participants’ impressions of it.*

**Response:** I totally agree, but things are not so simple. I have actually tried to find local medical doctors to collaborate, or a local hospital, with no success. I have been working with these models for almost a decade now, yet I have never had the opportunity to work directly with medical doctors, except when they were already researchers themselves. To test the app, in addition to ethical and bureaucracy issues (and there are many), I need to find medical doctors with patients and who are friendly with those tools. According to my experience, except for medical doctors that are researchers, they do not perceive these tools well; a lack of information is one reason, a culture that is still immature is another. Some seem to see them as competitors (the models against medical doctors) and overlook the models since they make mistakes (Daniel Kahneman stressed that in his last book). This is something that has already been discussed in the literature. I have added a new section with those comments in the new version. I hope that helps. I totally agree, and I would love to collaborate with any interested researchers and with medical doctors and patients interested in testing the system.

*3. It is important to also include a section discussing the potential dangers and ethical implications of deploying such a chatbot in the real world given the sensitive context and its critical implications.*

**Response:** This is not a straightforward discussion, but I have added a section on that. This section is best done in papers dedicated to the topic. During my PhD, I was working with white box models, and this discussion is also present in this area of applied mathematics. I have added one section in the Discussion section.

### Anonymous [3]

**Author’s note:** I am almost certain this reviewer did not receive the attachments. I have spent almost one month going back and forth with the support team to make the attachments available alongside the main paper. The paper was online without the attachment. I have decided to neglect answering this reviewer’s questions when it is evident the attachment would have solved the issue, but see my replies back. The information is in the attached material.

*1. I was really liking the idea of this paper and read it with great interest, but perhaps I misunderstood—I was hoping it was a chatbot that would actually give me a diagnosis (eg, once I input an image of my retina or have some conversation with it) rather than just referring me to a specialist (which would be appropriate in some cases). Please correct my understanding if I am wrong.*

**Response:** Yes, the system is a chatbot to which you can upload images as well as information like glucose levels. The system works both with images and physiological information. The chatbot uses large language models to extract the information from text or read images with an image description. Referring the user to a specialist is a last-stage trick, a bonus. I should stress: this is a prototype, a test of concept. Thus, the system should be seen as such, not as a production-ready chatbot. Therefore, the first impression of the reviewer was correct, though I am not sure he/she read the paper completely, since the paper does what he/she thought. It is curious why he/she got this wrong impression; maybe just read the conclusions or sections of the paper.

Would it be possible this reviewer did not receive the attachments? I have spent almost one month with the support team to fix this issue; the paper was uploaded for open review without the attachments. Finally, they updated the paper with attachments, almost one month later. My fear was a review of this type; the examples of the chatbot are in the attachments. This would explain the comments of the reviewer.

*2. From the initial paper idea, I got the feeling that it was going to be an app where I could start uploading images of X-rays and the chatbot, using its models, would start to tell me something about the image; instead, it seems from the example figures that all it is doing is telling the user that this is an X-ray image and to contact an expert.*

**Response:** Yes, that is correct! Again, it is possible the reviewer did not receive the attachment, where there are examples of how the chatbot behaves.

There are several examples in the attachment; it makes a diagnosis and also can make conversation, give extra information, and more. One limitation is that I have not focused on making an open conversation chatbot; it is easy to do so, I just need to create a memory for the chatbot. I have already done that for other projects, and it works just fine.

*2. Perhaps I read the paper in haste and am lacking understanding.*

**Response:** It is also possible the reviewer did not read the paper with care and attention.

*2. I would suggest showcasing a full conversation from each of your areas (X-rays, diabetes, etc), with a full screen capture of the conversation, showing an image uploaded and ending with a diagnosis (if indeed that is what your bot is capable of).*

**Response:** Again, the reviewer may not have received the attachment. I have shown one conversation per case, one by one, with extra discussions.

I should say one thing. The app is functional, but after I announced it on Product Hunt [4], it got interest fast. This means that I had to pay OpenAI for each interaction. I even had an application programming interface (API) key that leaked. I had to put the app offline since I pay those usage fees on my own. The idea is not a production software, but rather a proof of concept. I wanted to show it works. If the reviewer lets me know when they plan to use the app, I can make it available for one week, fully functional. If the reviewer sends me an API key of their own, it can stay online as long as they want.

Here is a review from Fibaly Group:

Hey there, my friend! Talk about making doctor visits a little more fun and less intimidating. I’m not sure how they do it, but kudos to @ideacodinglab for creating such a clever and unique product! Keep up the awesome work!

*3. Figures 2-5 do not really give me any picture of what is going on, they just reaffirm what I thought; that is, that the bot is not actually giving any information except recognizing what type of image it is, then referring the user to a consultant? Is that correct? You really need to put some nice figures of your full flow and architecture, not too complex, but the ones you show do not really, in my opinion, provide the reader with any real value.*

**Response:** We should keep in mind that the bot is using an API from OpenAI, which is doing most of the work as a large language model. Not sure it is my job to describe their API as they have documentation. Not sure what more I can add to the diagrams already created. The bot is a front end–like system. It just unites different techniques, that is, specialized models with the latest OpenAI API releases. The details of each model are either from OpenAI or from the user that created the specialized models. Thus, it makes no sense for me to add details of what each model is doing. I have added details in the supplementary materials for the models I have trained.

*3. As a reader, I want to see what it is you have done, and as a technical person who wants to replicate your work, I would want to see your architecture in diagram form, as well as a proper flowchart of some sort (again, no need to be complex, but to a high standard as you normally see in leading journals) outlining exactly what the flow is; this should correspond to the actual app screen captures so readers can see exactly what your app does.*

**Response:** I have already provided several diagrams. Giving further details would make it more like a tutorial, rather than an original research paper. I was asked to shorten the paper; the requested change would certainly increase the word count to more than 10,000 words.

*4. Having worked on and researched chatbots, I read this with great interest but, as per my comments, I am a little confused, as it seems this bot simply refers the user to a person after recognizing an image as an X-ray, for example. I was under the impression from the content or was half expecting the ability to input an image, be that of an X-ray or retina, and it would start giving me some diagnostic information or the like.*

**Response:** Yes, it does. See the examples in the attachment or let me know when you are planning to use the app. I can make it available for testing for one week, with my own key, at my own cost. I could create a user section, but it will take a while to do that.

## Round 2 Review

### Anonymous [1]

#### General Comments

*Thank you to the author for reading our comments and revising the paper. Many of our previous comments have been addressed; however, I still believe the writing style, grammar, and language of the paper need significant work before this can be published.*

**Response:** Thank you for your detailed and constructive feedback. I understand the concern regarding the language and style. For this revised version, I have taken additional steps to improve clarity, grammar, and overall readability while still not changing the paper too much.

I have produced a version using large language models, which are very good at writing. I still hold that the current version is scientifically well-written, but if the editorial team decides that the text needs more proofreading, I would like to ask about the possibility of using large language models as they are free and very good at what they do. I use the latest version of ChatGPT (GPT-4). I have been using it for a while for my manuscripts, both in English and Portuguese, and it does an excellent job.

As I am applying for a full article processing charge waiver and cannot afford professional copyediting services, I have leveraged advanced language models to assist with improving the manuscript, and it would be possible to do this again. These tools have significantly evolved and can now provide high-quality formal writing support, which I have also successfully applied to previous publications. I believe the current version reflects a substantial improvement and hope it meets the journal’s standards.

### Anonymous [3]

#### General Comments

*The author has given a response to each point and I am satisfied.*

**Response:** Thank you. I appreciate the positive feedback. I am glad the revised version addressed your concerns and met your expectations.

## Abbreviations

API: application programming interface
